# A Case of Exophthalmic Goitre

**Published:** 1879-03-20

**Authors:** John A. Liddell

**Affiliations:** N. Y.


### *A CASE OF EXOPHTHALMIC GOITRE.*

BY JOHN A. LIDDELL, M.D., OF N. Y.

This disease is by no means common, and therefore the publication of the following case may do some good. The notes were taken at the time. Miss C., a maiden lady in good circumstances, about 34 years old, of stout build, dark hair, dark eyes, and brunette complexion, whose home was in the country, applied to me for treatment *March* 15, 1873. She has a large bilobate tumor in the throat, obviously developed in connection with the thyroid gland; the right lobe considerably larger than the left. Eyes also much protuberant, giving her a strange, staring appearance, with pupils somewhat dilated, but respondent to light. Says she first noticed the swelling in her throat about three years ago. During the last year, however, it has grown very rapidly. First noticed that her eyes were becoming too prominent about one year ago. The protrusion is increasing, and at times the eyeballs feel as if they would burst out of her head. Then she gets relief by pressing them back into the orbits with her fingers.

As to the goitre, it sometimes is considerably larger than at others; it sometimes, also, gives rise to choking sensations and difficult breathing, or suffocative attacks, with dread of impending death. Is very nervous and easily fatigued. Countenance and lips rather pale. Is subject to dizziness, and is short-breathed, especially on making exertion. Is *not* liable to palpitation of the heart. Menstruation regular, much more so than formerly. Appetite good. Bowels inclined to be constipated. Suffered much from bleeding piles last fall, but of late not at all. Pulse about 90, full and hard. Heart beats strongly, but without abnormal murmurs. On examining the goitre with my fingers I find that each lobe of it pulsates synchronously with the heart, distinctly, strongly, and expansively, *i. e.*, in an outward direction, and imparts to the touch a jarring or thrilling sensation. On listening to it with a flexible stethoscope, a rather loud bellows-murmur is heard in every part of each lobe. Advised the application of ice to the goitre daily, and the internal administration of the following:

℞ Pulv. digitalis (opt.).........gr. xij.
Ferri redacti (Quevenne)........gr. xviij.
Mucilag. g. tragacanth..........q. s.
Ut ft. pil. æqual. No. 18.
S. Take one pill three times a day, before meals.

Also:

℞ Pil. rhei comp. (sugar-coated), No. 6.
S. Take one pill daily after dinner, until the constipation is relieved.

*March* 21. Patient feels rather better, and is less nervous; has not had an attack of choking since she called on the 15th; goitre unchanged; has not applied the ice; pulse 90 (by count) and soft. Prescribed the following:

℞ Extract. ergot. fluid. (Squibb).
Tinct. digitalis............ aa ℨ iij
S. Take ten drops three times a day, in water.

Also:

℞ Pil. rhei comp. (sugar-coated), No. 6.
S. Take one pill daily at bed time.

*March* 29. Patient says she is less nervous; finds now that she can write without shaking; exophtahlmos the same; pulse 90, by count, but larger; goitre smaller; has much pain in uterine, sacral, and lumbar regions, apparently due to the ergot; says that on the whole she feels decidedly stronger and better. Applied the ice yesterday to the goitre, which, she thinks, caused it to shrink, but gave her a severe fit of suffocative breathing that followed the application thereof. Ordered the ice to be applied again on Monday (31st), and to call on me Tuesday. Also:

℞ Pulv. digitalis (opt.)
Quiniæ sulph............. aa gr. xxiv.
Mucil. g. tragacanth....... q. s.
Ut ft. pil. æqual. No. 24.
S. Take one pill three times a day.

*April* 3. Patient says she feels still better; is decidedly stronger and less nervous; expression of countenance less anxious and more composed; eyes rather less protuberant; pulse less frequent and more natural in volume; after resting it is 84, by count; the goitre, especially its right lobe, decidedly diminished in size, and firmer in feel; bellows-murmur in right lobe also not so loud. Injected subcutaneously over right lobe of the goitre six minims of Squibb's extract. ergot. fluid. It caused some burning pain at the place of injection, but not so much as I had expected. Directed the pills of digitalis and quinine to be continued. She had failed or neglected to apply the ice again, partly from want of facilities to do it, and partly from dread of its possible effects, inasmuch as on a previous occasion it was attended with a paroxysm of choking and extreme dyspnœa.

*April* 25. Both lobes of the thyroid (goitre) now free from thrill, quite hard, and considerably diminished in size. Prescribed:

℞ Granulæ acid. arseniosi (sing. gr. 1-20, No. 100)
S. Take one granule night and morning.

On the next day she returned to her home in the country feeling pretty well, since which time I have not seen her. I heard, however, through one of her friends who had seen her, that the improvement was permanent.

What struck me most in the management of her case was the good effect of the ergot, especially when it was employed subcutaneously at the seat of the swelling in her throat.

I have delayed the publication of her case because, hitherto, I have hoped to see her again, and to find by personal examination, the result of the treatment.—*N. Y. Med. Record.*

